# Adenovirus and the Cornea: More Than Meets the Eye

**DOI:** 10.3390/v13020293

**Published:** 2021-02-13

**Authors:** Jaya Rajaiya, Amrita Saha, Ashrafali M. Ismail, Xiaohong Zhou, Ting Su, James Chodosh

**Affiliations:** Massachusetts Eye and Ear, Harvard Medical School, 243 Charles Street, Boston, MA 02114, USA; jaya_rajaiya@meei.harvard.edu (J.R.); amrita_saha@meei.harvard.edu (A.S.); mohamed_ismail@meei.harvard.edu (A.M.I.); xiaohong_zhou@meei.harvard.edu (X.Z.); ting_su-mail@meei.harvard.edu (T.S.)

**Keywords:** adenovirus, epidemic keratoconjunctivitis, human corneal epithelium, viral receptor

## Abstract

Human adenoviruses cause disease at multiple mucosal sites, including the respiratory, gastrointestinal, and genitourinary tracts, and are common agents of conjunctivitis. One site of infection that has received sparse attention is the cornea, a transparent tissue and the window of the eye. While most adenovirus infections are self-limited, corneal inflammation (keratitis) due to adenovirus can persist or recur for months to years after infection, leading to reduced vision, discomfort, and light sensitivity. Topical corticosteroids effectively suppress late adenovirus keratitis but are associated with vision-threatening side effects. In this short review, we summarize current knowledge on infection of the cornea by adenoviruses, including corneal epithelial cell receptors and determinants of corneal tropism. We briefly discuss mechanisms of stromal keratitis due to adenovirus infection, and review an emerging therapy to mitigate adenovirus corneal infections based on evolving knowledge of corneal epithelial receptor usage.

## 1. Introduction

The human adenovirus (HAdV) is a ubiquitous pathogen that has contributed much to current knowledge of molecular biology, leading to critical revelations about the cell cycle and RNA splicing, among other discoveries [1]. Infections with HAdV are a significant source of morbidity and mortality, world-wide and at all ages, through readily transmittable infections at mucosal sites [2]. Infection may be especially lethal in infants [3,4,5,6] and the immune compromised [7,8,9,10], but can also cause fatal acute respiratory distress syndrome in healthy adults [11,12]. HAdVs segregate phylogenetically into seven species (A–G), with 104 types [13,14]; 73 of the 104 fall within species D. The major corneal pathogens, all within species D, are HAdV-D8, 37, 53, 54, 56, 64 (previously typed as 19a), 82, and 85 (the latter two recently emerged) [15,16,17,18,19,20,21,22,23,24,25]. In this brief review, we discuss mechanisms of infection of the corneal epithelium by HAdVs, the immunologic manifestations of corneal infection, and recent approaches to antiviral therapy.

## 2. Adenoviral Eye Infection

HAdV infections of the eye are common, and the most frequent cause of viral conjunctivitis [26,27]. HAdV ocular infections manifest as either simple follicular conjunctivitis, pharyngoconjunctival fever, or epidemic keratoconjunctivitis (EKC). The first two are self-limited and do not involve the cornea. In contrast, corneal involvement is the sine qua non of EKC, and can become chronic and/or recurrent in ¼–½ of cases [28,29], with no specific therapy available [22]. The clinical signs of acute EKC include follicular conjunctivitis and preauricular lymphadenopathy, both common to other viral infections, but with a more explosive clinical course [30,31]. The contralateral eye in EKC is affected in ~70% of cases [32]. Inflammatory conjunctival membranes form in ¼–½ of those with EKC [28,31]; if untreated, these membranes become incorporated into host tissue and can form scars that may restrict eye movement and/or cause symptoms of dry eye [33].

Conjunctival membrane formation and keratitis distinguish EKC from other ocular adenovirus infections [31,34,35]. The epithelial keratitis in EKC presents within the first few days of symptoms with either punctate or large, geographic-shaped epithelial erosions [34], and typically resolves in several days. However, stromal keratitis then ensues—in 60% of cases in a recent large study [21]. Characteristic multifocal, subepithelial, leukocytic infiltrates appear in the corneal stroma at 14–21 days after onset of the clinical signs of infection [21]. These infiltrates most commonly resolve within a few weeks of onset, but in a significant proportion of persons will persist or recur for months to years [28,29,36]. In one study, 47% still had signs of stromal keratitis 2 years after onset of infection [29]. Adenovirus-associated stromal keratitis leads to foreign body sensation and symptoms of glare, irregular astigmatism, and blurred vision [37], and can cause permanent corneal scarring [38].

## 3. The Cornea Facsimile

Animal models to study adenoviral pathogenesis have been limited by the specificity of HAdVs for human cells; mouse models can be used only to study innate immune responses to the virion [39,40,41]. To determine the pathogenesis of adenovirus stromal keratitis, Rajaiya and coworkers [42] modified a previously described 3-D in vitro model of the human cornea, the “human corneal facsimile” [43]. In this model, primary cultured human keratocytes (the fibroblast-like resident cells of the corneal stroma) were mixed with type I collagen in 3-μm pore size transwell inserts, and overlayed with Matrigel^®^ [44], an epithelial basement membrane-like layer, in order to simulate a human corneal stroma and epithelial basement membrane, respectively. When infected from the (upper) Matrigel side with HAdV-D37, CXCL8 expressed by infected keratocytes co-localized with heparan sulfate within the Matrigel in a multifocal pattern. When leukocytes derived from human peripheral blood were placed in the media beneath the inserts, neutrophils migrated upwards, against gravity, to foci of CXCL8 deposition, mimicking the subepithelial corneal infiltrates characteristic of human patients with EKC. These data support a mechanism for stromal keratitis in EKC in which infected keratocytes express chemokines that deposit at negatively charged moieties in the corneal epithelial basement membrane. However, evidence to support infection of corneal stromal cells in the intact human cornea by adenovirus during EKC is lacking. Regardless, successful replication of HAdV in the corneal epithelium in the early stages of EKC is very likely required for the later development of stromal keratitis [31,45].

## 4. Corneal Epithelial Cell Tropism and Receptors

An abundance of evidence supports corneal epithelial cell infection by HAdVs. In a large clinical series of EKC cases (*n* = 92), Imre and colleagues showed typical adenovirus inclusions in 85% of corneal epithelial scrapings of studied patients [46]. Dawson et al. then reported adenovirus-like particles within corneal epithelial cells from scrapings taken from a patient with EKC who was culture positive for HAdV-D8 [31]. Maudgal used impression cytology of the corneal epithelium in 12 patients with EKC, all culture positive for adenovirus, to show typical intracellular virus inclusions [45]. Chodosh and coworkers reported positive cultures for adenovirus from corneal scrapings obtained from six patients with epithelial keratitis [34]. They further showed that HAdV-D8, the most common cause of EKC world-wide [21], can replicate in primary human corneal epithelial cells cultured in vitro [34].

The tropism of particular HAdVs for corneal epithelial cells could account for the limited number of HAdV types associated with EKC [21]. However, Xiao and coworkers [47] successfully infected an SV40 immortalized corneal epithelial cell line derived by Araki-Sasaki and colleagues [48] with numerous different HAdV types, including some not typically associated with EKC. In this cell line, HAdV-C2, not known to cause EKC, grew to considerably higher titers than HAdV-D64, a common EKC pathogen. However, the opposite result—greatly favoring HAdV-D64 replication, was seen upon infection of whole human donor corneas in organotypic culture, and also when the cells were cultured on a vitronectin substrate. These data suggested that the SV40 immortalized cells grown on standard tissue culture ware do not adequately recapitulate human corneal epithelium in vivo. In contrast, experimental infections of a line of human corneal epithelial cells immortalized by transfection of human telomerase reverse transcriptase (hTERT) [49] more closely matched known clinical associations of specific types with EKC. In those studies, hTERT-immortalized human corneal epithelial cells were permissive of viral replication only for those types associated with EKC, while not permitting infection by types not associated with EKC [17,18,50].

As with all mucosa, the eye is bathed in secretions with antiviral components, [51] and the preocular tear film must be successfully negotiated by any pathogen for infection to occur. Menon and coworkers also showed a role in HAdV tropism for the membrane associated mucin-rich glycocalyx covering the apical surface of the corneal epithelium [52]. The cornea-tropic HAdV-D37, but not HAdV-D19, induced the release of MUC16 ectodomain in corneal epithelial cells [52], and this reduced the barrier to corneal epithelial cell infection. The preocular tear film may also enhance [53] or inhibit [54] adenoviral entry, the latter reportedly mediated by glycosaminoglycans in tears. Suranim, a glycosaminoglycan mimetic, inhibited the attachment of HAdV-D37 to SV40-immortalized human corneal epithelial cells, and was predicted to also block attachment of other EKC-associated viruses within HAdV-D [55].

The primary ligand for adenovirus binding to its target cell is the trimeric fiber protein [56,57]. The fiber extends outward from each penton base capsomer, located at the 12 apices of the icosahedral capsid. The most distal component of the fiber, the “knob”, has been mapped for specific sites of receptor binding [56,58,59,60,61,62,63,64,65,66,67] to several cell surface molecules. The specific receptor utilized depends on the structure and amino acid signatures displayed on the fiber knob [59,68], and has impact downstream on intracellular trafficking and inflammation [69,70]. Receptors previously reported for HAdVs include the Coxsackie-Adenovirus receptor [56,71], sialic acid [72,73,74,75], GD1a glycan [58], CD46 [76,77], desmoglein 2 [78], and other constitutive cell surface molecules [79,80,81]. Both GD1a glycan and CD46 were previously reported to bind those HAdVs associated with EKC [58,72,73,75,82,83].

**Figure 1:**
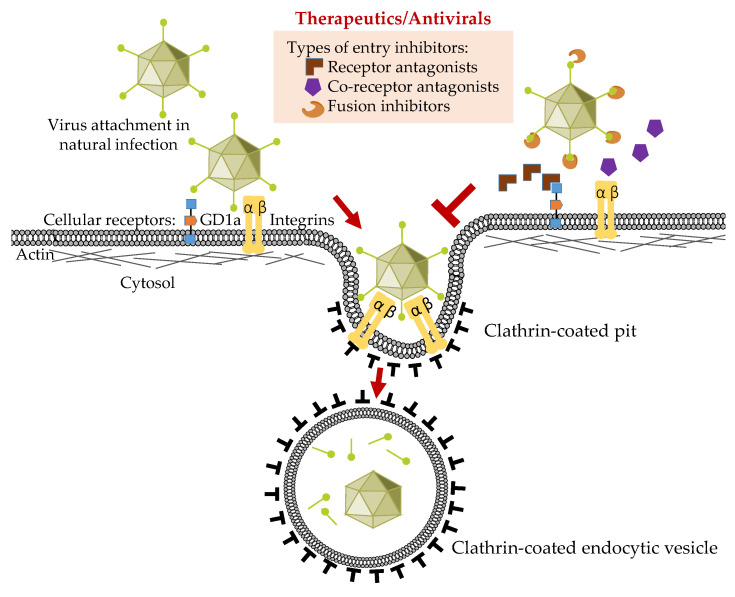
Schematic representation of the adenovirus entry process in corneal epithelial cells and possible viral entry inhibitors.

Recent evidence has further clarified receptor-mediated entry of those types within HAdV-D associated with EKC, resulting in the first potential viral entry-based therapy for acute infection (Figure 1). As discussed briefly above, it was shown that HAdV-D37 binds to sialic acid [72,73,74,75], and that binding in vitro to corneal epithelial cells can be inhibited by multivalent sialic acid conjugates [84]. More recently, it was demonstrated that HAdV-D37 binds specifically to two sialic acids present on GD1a glycan [58]. These studies used the same SV40 immortalized corneal epithelial cell line [48] discussed above. Subsequent work showed that some but not all of the EKC-associated HAdVs bind to sialic acid-expressing cell surface glycans [85], and that various multivalent sialic acid constructs could block binding [84,86,87,88,89,90,91]. Sites on the fiber knob that bind to the various potential adenovirus receptors have been mapped in exquisitely detailed structural studies [92]. Binding is not only specific to HAdV species [76], but also differs within HAdV species [65,77,93,94]. A recent study of the fiber knobs of HAdV-D revealed a distinct phylogenetic clade encompassing those associated with EKC [95]. Specifically, an association with EKC, and by inference with corneal epithelial cell tropism, was predicted by a lysine or alanine at amino acid residue 240 on the knob.

In the most widely cited paradigm for adenoviral entry (Figure 1), primary binding of the fiber knob to its receptor is followed by secondary contact between an arginine-glycine-aspartic acid (RGD) motif in each penton base protein with host cell integrins including α_v_β_3_, α_v_β_5_, and α_v_β_1_ [96,97,98] (and for human corneal epithelial cells, α_v_β_1_ and α_3_β_1_ [98]). This in turn induces aggregation of the integrins [99,100], which results in changes in the integrin conformation [101] and autophosphorylation [102], and leads to activation of focal adhesion kinase [103,104] and Src kinase [105,106]. These changes in intracellular signaling in turn activate clathrin-mediated endocytosis [103,107,108]. For the non-EKC-associated HAdV-D9, the fiber is relatively short and allows direct interaction with host cell integrins without fiber knob/receptor engagement [74,109]. Recently, the hexon protein of the EKC pathogen, HAdV-D56 [17], was shown to bind directly to CD46; soluble CD46 also inhibited binding of 16 out of 17 HAdV-D types tested [110]. To add complexity to this story, CD46 binding has been associated specifically with internalization by clathrin-coated pits, but when cross-linked by a multivalent ligand, CD46 binding can lead to entry by macropinocytosis [111].

Although the mechanistic connection between binding of the adenovirus fiber knob to a cell surface receptor and secondary aggregation of cell membrane integrins is relatively clear, the molecular events that connect integrin aggregation to endocytosis have not been clarified in corneal epithelial cells. Lee and coworkers recently showed that entry of HAdV-D37 occurs by a noncanonical clathrin-mediated entry pathway in both hTERT-immortalized human corneal epithelial cells, and in primary cultured human corneal epithelial cells [112]. More studies are needed, but in human keratinocytes, α_v_β_5_ can cluster in clathrin-enriched foci [113]. Aggregation of integrin β3 by RGD motifs can recruit clathrin endocytic machinery [114], and clathrin can mediate endocytosis of integrins for recycling [115]. These findings suggest future avenues of research.

In summary, existing evidence implicates corneal epithelial cells as key to adenoviral corneal pathogenesis, with likely contributions from keratocytes and resident bone marrow derived cells in the corneal stroma to the immunopathology of EKC. Recent discoveries related to corneal epithelial cell entry by HAdV-Ds suggest hope for the development of an effective therapy to mitigate immunopathology in EKC.

## Data Availability

No new data were created or analyzed in this study. Data sharing is not applicable to this article.
